# Joint effect of ischemic stroke and obesity on the risk of venous thromboembolism: the Tromsø Study

**DOI:** 10.1016/j.rpth.2024.102392

**Published:** 2024-03-27

**Authors:** Birgitte G. Tøndel, Joakim K. Sejrup, Vânia M. Morelli, Maja-Lisa Løchen, Inger Njølstad, Ellisiv B. Mathiesen, Tom Wilsgaard, John-Bjarne Hansen, Sigrid K. Brækkan

**Affiliations:** 1 Thrombosis Research Group, Department of Clinical Medicine, UiT The Arctic University of Norway, Tromsø, Norway; 2 Thrombosis Research Center, Division of Internal Medicine, University Hospital of North Norway, Tromsø, Norway; 3 Epidemiology of Chronic Diseases Research Group, Department of Community Medicine, UiT The Arctic University of Norway, Tromsø, Norway; 4 Brain and Circulation Research Group, Department of Clinical Medicine, UiT The Arctic University of Norway, Tromsø, Norway; 5 Department of Neurology, University Hospital of North Norway, Tromsø, Norway

**Keywords:** body mass index, ischemic stroke, obesity, pulmonary embolism, risk factors, venous thromboembolism, venous thrombosis

## Abstract

**Background:**

Patients with ischemic stroke have increased risk of venous thromboembolism (VTE). Obesity is prevalent in stroke patients and a well-established risk factor for VTE. Whether obesity further increases the VTE risk in patients with stroke remains unclear.

**Objectives:**

We investigated the joint effect of ischemic stroke and obesity on the risk of incident VTE in a population-based cohort.

**Methods:**

Participants (*n* = 29,920) were recruited from the fourth to sixth surveys of the Tromsø Study (1994-1995, 2001, and 2007-2008) and followed through 2014. Incident events of ischemic stroke and VTE during follow-up were recorded. Hazard ratios (HRs) of VTE with 95% CIs were estimated according to combined categories of ischemic stroke and obesity (body mass index ≥ 30 kg/m^2^), with exposure to neither risk factors as reference.

**Results:**

During a median follow-up of 19.6 years, 1388 participants experienced ischemic stroke and 807 participants developed VTE. Among those with stroke, 51 developed VTE, yielding an incidence rate of VTE after stroke of 7.2 per 1000 person-years (95% CI, 5.5-9.5). In subjects without stroke, obesity was associated with a 1.8-fold higher VTE risk (HR, 1.76; 95% CI, 1.47-2.11). In nonobese subjects, stroke was associated with a 1.8-fold higher VTE risk (HR, 1.77; 95% CI, 1.27-2.46). Obese subjects with stroke had a 2-fold increased VTE risk (HR, 2.44; 95% CI, 1.37-4.36).

**Conclusion:**

The combination of obesity and ischemic stroke did not yield an excess risk of VTE. Our findings suggest that obese subjects with ischemic stroke do not have a more than additive risk of VTE.

## Essentials

• Ischemic stroke (IS) is associated with an increased risk of venous thromboembolism (VTE).
• Obesity may further increase the VTE risk in patients with IS.
• We investigated the combined effect of IS and obesity on VTE risk in a large cohort.
• Obesity did not result in an excess risk of VTE in patients with IS.

## 1 Introduction

Over the last 2 decades, several studies have shown that patients with ischemic stroke are at increased risk of developing venous thromboembolism (VTE), clinically manifesting as pulmonary embolism (PE) or deep vein thrombosis (DVT) [[1], [2], [3], [4]]. In population-based studies, risk estimates for VTE have been reported to be particularly high during the first few months after the stroke event and decline rapidly thereafter [[2], [3], [4]]. For instance, in the Tromsø Study, a 20- and 11-fold increased risk of VTE was reported in the first and subsequent 2 months after ischemic stroke, respectively [2]. VTE development complicates stroke recovery and is associated with greater disability and lower survival rates [5,6].

The underlying mechanism for the association between ischemic stroke and VTE is not fully understood as there are limited data on explanatory factors [7]. The transient and short-term nature of the increased VTE risk following an acute stroke event [[2], [3], [4]] indicates that mechanisms or conditions related to the stroke itself contribute substantially to the risk of VTE. Indeed, a population-based case-crossover study found that immobilization and infection accounted for about two-thirds of the total effect of stroke on VTE risk [8]. Still, other factors could play a considerable role in the development of stroke-related VTE. Identification of risk factors that particularly increase the risk of VTE after stroke may improve risk stratification and guide clinical decisions on thromboprophylaxis and thereby reduce the burden of stroke-related VTE in the society.

Obesity is a well-established risk factor for VTE and is associated with a 2- to 3-fold increased VTE risk in the general population [9,10]. The prevalence of obesity is increasing dramatically worldwide [11], and obesity is a common condition in patients with ischemic stroke [12]. As obesity is associated with both venous stasis [9,13], low-grade inflammation, and hypercoagulability [14,15] as well as attenuated fibrinolysis [16,17], it could add to a hypercoagulable and inflammatory poststroke state [[18], [19], [20], [21], [22]], resulting in an excess risk of VTE in obese stroke patients. A few previous studies have assessed the impact of overweight and obesity on the risk of VTE among patients with ischemic stroke [[23], [24], [25]]. However, the studies were conducted within a prediction framework, outcomes were limited to DVT only, and obesity was not investigated according to commonly used clinical cutoffs. Thus, the role of obesity in the development of stroke-related VTE remains uncertain.

In previous studies, obesity has been shown to yield synergistic effects on the risk of VTE in combination with other risk factors, such as tall body stature [26], some prothrombotic genotypes [27,28], and use of oral contraceptives [27]. Moreover, we recently reported that the combination of myocardial infarction and obesity yielded a supra-additive effect on VTE risk, where 46% of the VTE events in the joint exposure group could be attributed to the combination of the 2 risk factors [29]. Therefore, the aim of this study was to investigate the joint effect of ischemic stroke and obesity on the risk of incident VTE in a large cohort study derived from the general population.

## 2 Methods

### 2.1 Study population

Study participants were recruited from the fourth, fifth, and sixth surveys of the Tromsø Study, which is a Norwegian, single-center, prospective, population-based study, with repeated health surveys of the inhabitants of the Tromsø municipality, where the vast majority of the inhabitants are of Caucasian origin. A detailed description of the Tromsø Study has been published elsewhere [30]. The Tromsø Study was approved by the Regional Committee for Medical and Health Research Ethics in Northern Norway, and all participants provided informed written consent to participate. In total, 30,288 individuals aged 25 to 97 years attended ≥1 of the 3 surveys that were conducted in 1994-1995 (Tromsø4), 2001 (Tromsø5), and 2007-2008 (Tromsø6). The overall attendance was high, with 77% in Tromsø4, 79% in Tromsø5, and 66% in Tromsø6. Participants not officially registered as inhabitants of the municipality of Tromsø at the date of study enrollment (*n* = 20), participants with a VTE or ischemic stroke event before the inclusion date (*n* = 86 and *n* = 218, respectively), and participants with missing data on body mass index (BMI, *n* = 44) were excluded. Thus, 29,920 unique study participants were included and followed from enrollment until end of follow-up (December 31, 2014) or the date of VTE, migration, or death, whichever occurred first (Figure).

**Figure:**
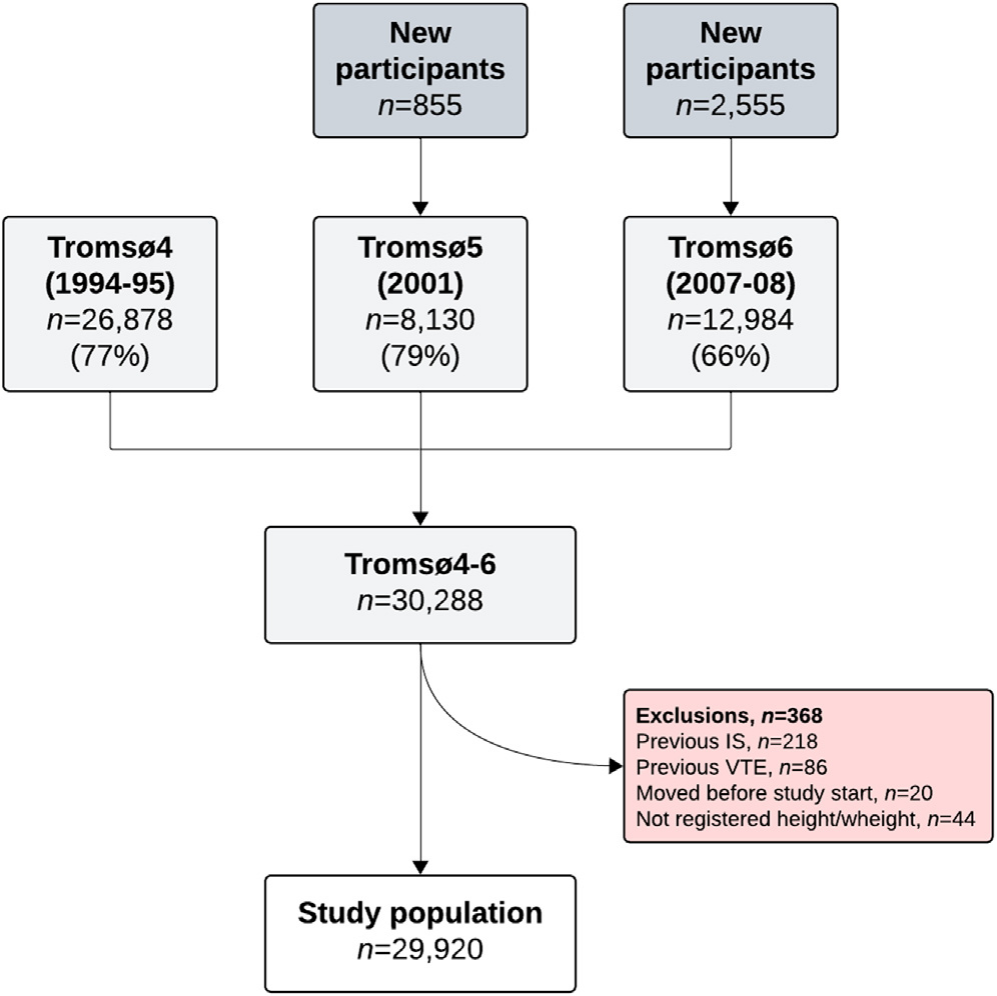
Inclusion of 29,920 unique study participants from the fourth (1994-1995), fifth (2001), and sixth (2007-2008) surveys of the Tromsø Study (Tromsø4-6). IS, ischemic stroke; VTE, venous thromboembolism.

### 2.2 Baseline measurements

At baseline in each survey, information was obtained from self-administered questionnaires, physical examinations, and blood samples. Definitions and measurement methods of baseline variables in the Tromsø Study have been described in detail elsewhere [30,31]. At the physical examination, body height and weight were measured with subjects wearing light clothing and no shoes. BMI was calculated as weight in kilograms divided by the square of height in meters (kg/m^2^). In line with the World Health Organization (WHO) classification [11], obesity was defined as BMI of ≥30 kg/m^2^. Absence of obesity was defined as BMI of <30 kg/m^2^. Blood pressure was measured 3 times with a digital automatic device, and the mean of the last 2 recordings was used in the analyses. Hypertension was defined as mean systolic blood pressure of ≥140 mmHg or mean diastolic blood pressure of ≥90 mmHg or self-reported use of antihypertensive medication. For lipid profiles, nonfasting blood samples collected from an antecubital vein were analyzed by standard methods at the University Hospital of North Norway (UNN). Hypercholesterolemia was defined as serum total cholesterol of ≥6.5 mmol/L. Information on diabetes mellitus and smoking habits was obtained from the self-administered questionnaires.

### 2.3 Exposure assessment: ischemic stroke

Ischemic stroke was defined according to the WHO definition when computed tomography or magnetic resonance imaging scans or autopsy had ruled out brain hemorrhage [32]. All incident events of ischemic stroke during follow-up were identified by linkage of the Norwegian national 11-digit identification number to the hospital discharge diagnosis registry at the UNN, as previously described [2]. For case validation, an endpoint committee reviewed in-hospital and out-of-hospital ischemic stroke events based on data from hospital and out-of-hospital medical records, autopsy records, and death certificates.

### 2.4 Confounder assessment: atrial fibrillation

Atrial fibrillation (AF) diagnosed before inclusion or during follow-up was identified by searching the discharge diagnosis registry at UNN, as previously described [33]. In brief, the diagnosis of AF had to be documented by an electrocardiogram, and an independent endpoint committee validated each case. For the present study, both paroxysmal and permanent forms were included in the definition of AF. Episodes of transient AF documented only in relation to acute cardiac disease (such as myocardial infarction) or cardiac or other surgery were not classified as AF events.

### 2.5 Outcome assessment: VTE

All incident VTE events during follow-up were identified by searching relevant hospital registries (radiology, discharge diagnosis, and autopsy) at UNN, as previously reported [34]. An endpoint committee reviewed medical records for case validation, and the validation criteria included clinical signs and symptoms of DVT or PE, combined with objective confirmation by a radiological procedure, which resulted in treatment initiation, unless contraindications were specified. VTE events from the autopsy registry were included when the death certificate indicated VTE as the cause of death or a significant condition associated with death. A VTE event was classified as either PE (with or without concurrent DVT) or isolated DVT. Further, the VTE events were classified as provoked or unprovoked based on the presence of provoking risk factors at the time of diagnosis. Immobilization (ie, bed rest for >3 days, wheelchair use, or long-distance travel exceeding 4 hours within the 14 days prior to the event), surgery or trauma within the prior 8 weeks, and active cancer were considered as provoking factors, in addition to any other potential provoking factor described by a physician in the medical record (eg, intravascular catheter). Acute medical conditions (ie, ischemic stroke, myocardial infarction, and acute infection) were not included in the definition of provoked VTE.

### 2.6 Statistical analysis

Participants who developed ischemic stroke during follow-up contributed with nonexposed person-time from the date of study entry to the date of a diagnosis of ischemic stroke and then with exposed person-time from the date of the stroke event and onwards. For each study participant, nonexposed and exposed person-years were counted from the date of inclusion to the date of an incident diagnosis of VTE, the date the participant died or moved from the municipality of Tromsø, or the end of follow-up (December 31, 2014), whichever occurred first. Participants who moved from the municipality or died during follow-up were censored at the date of migration or death. Statistical analyses were performed using STATA version 18.0 (Stata Corporation). Crude incidence rates (IRs) of VTE were calculated and expressed as number of events per 1000 person-years at risk. To assess the joint effect of ischemic stroke and obesity on the risk of incident VTE, Cox proportional hazards regression models were used to estimate hazard ratios (HRs) with corresponding 95% CIs for VTE according to categories of ischemic stroke exposure and obesity status using subjects with exposure to neither risk factors as reference group. In addition, the impact of obesity on VTE risk among patients with ischemic stroke was assessed using nonobese subjects with ischemic stroke as reference group. Ischemic stroke was included as a time-varying risk factor in the Cox regression models. Age was used as time scale in the Cox regression models, with the age of the participants at study enrollment defined as entry time and the age at the VTE event or censoring event (ie, death, migration, or end of follow-up) defined as exit time. HRs for VTE were estimated with 2 different models. The first model was adjusted for age (as time scale) and sex, while the second model was additionally adjusted for AF. Subgroup analyses were carried out according to VTE subtype (PE and DVT) and the presence of provoking factors. The proportional hazards assumption was tested using Schoenfeld residuals and found to be not violated.

The presence of biological interaction between ischemic stroke and obesity was evaluated on an additive scale by calculating the relative risk attributable to interaction (RERI) and the proportion attributable to interaction (AP) with corresponding 95% CIs for VTE and VTE subtypes [35,36]. In short, the RERI can be interpreted as part of the total effect on the outcome that is attributable to interaction, and the AP can be interpreted as the proportion of the joint effect that is due to interaction between 2 risk factors [35]. Values >0 indicate presence of positive interaction for both RERI and AP, ie, they indicate that the effect on the outcome of the joint exposure to the 2 risk factors is greater than the sum of the 2 separate effects [35,36].

## 3 Results

In total, 29,920 participants were recruited from the fourth, fifth, and sixth surveys of the Tromsø Study (Figure). During a median follow-up time of 19.6 years, 1388 (4.6%) participants were diagnosed with a first-time ischemic stroke. The baseline characteristics of the study participants with and without ischemic stroke are presented in Table 1. Patients with ischemic stroke were on average older than those without stroke (63 vs 46 years, respectively), and the stroke group had the highest proportion of males (55% vs 47%) as well as obese study participants (17% vs 11%).

**Table 1:** Baseline characteristics of study participants (*n* = 29,920) without and with ischemic stroke: the Tromsø Study, 1994-2014.

Baseline characteristics	No ischemic stroke (*n* = 28,532)	Ischemic stroke (*n* = 1388)
Age (y)	45.7 ± 14	63 ± 12
Sex (male)	47.1 (13,438)	54.5 (757)
BMI (kg/m^2^)	25.3 ± 3.9	26.5 ± 4.0
Obesity (BMI ≥ 30 kg/m^2^)	11.2 (3187)	17.4 (241)
Total cholesterol (mmol/L)	5.93 ± 1.29	6.70 ± 1.27
HDL (mmol/L)	1.49 ± 0.41	1.47 ± 0.42
Triglycerides (mmol/L)	1.53 ± 1.03	1.82 ± 1.09
Systolic blood pressure (mmHg)	133 ± 20	153 ± 25
Diastolic blood pressure (mmHg)	77 ± 12	87 ± 14
Hypertension^a^	32.7 (9333)	71.3 (989)
Hypercholesterolemia^b^	31.4 (8958)	54.9 (762)
Smoking^c^	35.9 (10,213)	34.5 (479)
Self-reported diabetes mellitus	1.6 (466)	6.1 (84)

Values are % (*n*) for categorical variables or mean ± SD for continuous variables.

BMI, body mass index; HDL, high-density lipoprotein.

a Mean systolic/diastolic blood pressure of ≥140 mmHg/≥90 mmHg or self-reported use of antihypertensives.

b Total cholesterol level of ≥6.5 mmol/L.

c Self-reported daily smoking: yes/no.

In our cohort, 807 patients developed incident VTE during follow-up, of which 51 events occurred in patients with ischemic stroke. This yielded an overall incidence rate of VTE after stroke of 7.2 per 1000 person-years (95% CI, 5.5-9.5). Clinical characteristics of the VTE events in study participants with and without ischemic stroke are reported in Table 2. The proportions of DVT and PE were similar in both groups. In the ischemic stroke group, the proportion of provoked VTE events was higher than that in the nonstroke group (73% vs 52%, respectively). Immobilization was the most frequent provoking factor in the stroke group (47% vs 19%, respectively). IRs and corresponding multivariable HRs of overall VTE, PE, and DVT according to ischemic stroke exposure and obesity status during follow-up are reported in Table 3. In subjects without ischemic stroke, obesity was associated with a 1.8-fold higher risk of VTE (HR, 1.76; 95% CI, 1.47-2.11) compared with the reference group. In nonobese subjects, ischemic stroke was associated with a 1.8-fold higher risk of VTE (HR, 1.77; 95% CI, 1.27-2.46). In the joint exposure group (ie, obese subjects with ischemic stroke), the risk of VTE was increased by 2.4-fold (HR, 2.44; 95% CI, 1.37-4.36) compared with the reference group. Subgroup analyses with PE and DVT as outcomes yielded essentially similar results as those for overall VTE. Of note, the increased thrombosis risk was slightly more pronounced for PE than for DVT, with HRs in the joint exposure groups of 2.94 (95% CI, 1.37-6.30) for PE and 1.98 (95% CI, 0.81-4.83) for DVT when compared with the reference group. Among study participants with ischemic stroke only, obesity was not associated with risk of overall VTE (HR, 1.37; 95% CI, 0.71-2.65) or DVT (HR, 1.05; 95% CI, 0.39-2.80), while the risk appeared to be increased for PE (HR, 1.79; 95% CI, 0.72-4.41), though with a very wide CI. In Table 4, measures quantifying interaction on an additive scale (ie, RERI and AP) for the joint effect of ischemic stroke and obesity on the risk of VTE and VTE subtypes are presented. Estimates of RERI and AP indicated a less than additive effect of the combination of ischemic stroke and obesity on the risk of overall VTE and DVT. For PE, the combination of ischemic stroke and obesity was suggestive of having a more than additive effect on the thrombosis risk, with a RERI of 0.41 (95% CI, −1.96 to 2.77) and an AP of 0.14 (−0.57 to 0.85), although not statistically significant. In Supplementary Table S1, IRs and corresponding multivariable HRs of unprovoked and provoked VTE according to ischemic stroke exposure and obesity status are reported. For unprovoked VTE, no association was observed in the joint exposure group (HR, 0.87; 95% CI, 0.21-3.51). Participants jointly exposed to obesity and ischemic stroke had a 4-fold (HR, 3.83; 95% CI, 2.02-7.27) increased risk of provoked VTE compared with the reference group, and measures of interaction (Supplementary Table S2) indicated a nonsignificant positive interaction between the 2 risk factors, with a RERI of 0.85 (95% CI, −1.72 to 3.43) and an AP of 0.22 (95% CI, −0.32 to 0.77).

**Table 2:** Characteristics of venous thromboembolism events (*n* = 807) by ischemic stroke status: the Tromsø Study, 1994-2014.

Characteristics of VTE events	No ischemic stroke (*n* = 756)	Ischemic stroke (*n* = 51)
Clinical characteristics		
Deep vein thrombosis	57.3 (433)	54.9 (28)
Pulmonary embolism	42.7 (323)	45.1 (23)
Provoked	52.4 (396)	72.6 (37)
Unprovoked	47.6 (360)	27.5 (14)
Provoking factors		
Surgery	15.9 (120)	11.8 (6)
Trauma	9.1 (69)	8.0 (4)
Cancer	24.3 (184)	19.6 (10)
Immobility^a^	19.2 (145)	47.1 (24)
Others^b^	4.8 (36)	2.0 (1)

Values are % (*n*).

a Bed rest for >3 days, journeys >4 hours by car, boat, train, or air within the last 14 days, or other types of immobilization.

b Other provoking factors described by a physician in the medical record (eg, intravascular catheter).

**Table 3:** Crude incidence rates and adjusted hazard ratios with 95% CIs of overall venous thromboembolism, deep vein thrombosis, and pulmonary embolism according to ischemic stroke exposure and obesity status: the Tromsø Study 1994-2014.

Ischemic stroke status	Obesity status	Person-years	VTE events	Crude IR^a^ (95% CI)	Joint effects^b^		Within IS group^c^	
					Model 1^d^ HR (95% CI)	Model 2^e^ HR (95% CI)	Model 1^d^ HR (95% CI)	Model 2^e^ HR (95% CI)
VTE								
IS −	Obesity −	400,129	601	1.5 (1.4-1.6)	Reference	Reference		
IS −	Obesity +	46,745	155	3.3 (2.8-3.9)	1.71 (1.43-2.04)	1.76 (1.47-2.11)		
IS +	Obesity −	5749	39	6.8 (5.0-9.3)	1.68 (1.20-2.33)	1.77 (1.27-2.46)	Reference	Reference
IS +	Obesity +	1291	12	9.3 (5.3-16.4)	2.16 (1.21-3.84)	2.44 (1.37-4.36)	1.27 (0.66-2.44)	1.37 (0.71-2.65)
PE								
IS −	Obesity −	400,129	251	0.6 (0.6-0.7)	Reference	Reference		
IS −	Obesity +	46,745	71	1.5 (1.2-1.9)	1.86 (1.42-2.42)	1.88 (1.44-2.46)		
IS +	Obesity −	5749	17	3.0 (1.8-4.8)	1.61 (0.97-2.65)	1.65 (1.00-2.72)	Reference	Reference
IS +	Obesity +	1291	7	5.4 (2.6-11.4)	2.77 (1.30-5.92)	2.94 (1.37-6.30)	1.65 (0.67-4.07)	1.79 (0.72-4.41)
DVT								
IS −	Obesity −	400,129	350	0.9 (0.8-1.0)	Reference	Reference		
IS −	Obesity +	46,745	84	1.8 (1.5-2.2)	1.61 (1.26-2.04)	1.68 (1.32-2.13)		
IS +	Obesity −	5749	22	3.8 (2.5-5.8)	1.74 (1.12-2.70)	1.87 (1.20-2.91)	Reference	Reference
IS +	Obesity +	1291	5	3.9 (1.6-9.3)	1.65 (0.68-4.02)	1.98 (0.81-4.83)	0.97 (0.36-2.58)	1.05 (0.39-2.80)

IS +/− indicates incident IS/no incident IS during follow-up, respectively. Of note, IS was included as a time-varying risk factor in the Cox regression models. Obesity +/− indicates body mass index ≥/< 30 kg/m^2^ at baseline, respectively.

DVT, deep vein thrombosis; HR, hazard ratio; IR, crude incidence rate; IS, ischemic stroke; PE, pulmonary embolism; VTE, venous thromboembolism.

a Per 1000 person-years.

b Joint effects of IS and obesity using nonobese subjects without IS as the reference group.

c Impact of obesity in subjects with IS, using nonobese subjects with IS as the reference group.

d Model 1: age as time scale, adjusted for sex.

e Model 2: Model 1 + atrial fibrillation.

**Table 4:** Measures of biological interaction with 95% CIs for the joint effect of ischemic stroke and obesity (body mass index ≥ 30 kg/m^2^) for overall venous thromboembolism, pulmonary embolism, and deep vein thrombosis: the Tromsø Study 1994-2014.

Outcome	Relative excess risk attributable to interaction (95% CI)	Proportion attributable to interaction (95% CI)
VTE	−0.09 (−1.61 to 1.43)	−0.04 (−0.68 to 0.61)
PE	0.41 (−1.96 to 2.77)	0.14 (−0.57 to 0.85)
DVT	−0.57 (−2.51 to 1.39)	−0.29 (−1.50 to 0.93)

DVT, deep vein thrombosis; PE, pulmonary embolism; VTE, venous thromboembolism.

## 4 Discussion

In this population-based cohort study, we investigated the joint effects of ischemic stroke and obesity on the risk of incident VTE. We found that obese individuals with ischemic stroke had a 2.4-fold higher risk of VTE compared with individuals exposed to neither risk factors, but the combined effects of the 2 exposures did not exceed the sum of the separate effects. Our findings suggest no biological interaction between ischemic stroke and obesity on the risk of incident VTE. Subgroup analyses with PE and DVT as outcomes yielded essentially similar results as those for overall VTE. In the general population, obesity is considered as a major and causal risk factor for VTE, and the risk of VTE increases linearly with increasing BMI [9,10,37]. Obesity has been shown to biologically interact with other risk factors to yield supra-additive effects on the risk of VTE [[26], [27], [28], [29]]. Further, ischemic stroke is a strong risk factor for VTE [2]. However, in the present study, the combination of ischemic stroke and obesity did not result in an excess risk of VTE, which suggests that the effect of the ischemic stroke itself or presence of other strong VTE triggers in relation to the stroke supersedes the effect of obesity. Findings from several studies support the notion that stroke-related factors are the main contributors to the development of VTE after stroke. The VTE risk following acute ischemic stroke has consistently been shown to have a transient and short-term nature, with rapidly declining risk estimates after the first few months [[2], [3], [4]]. Patients with ischemic stroke are hospitalized and usually immobilized for a time due to bed rest and/or leg paralysis, resulting in an increased susceptibility for thrombus formation because of venous stasis [38]. Accordingly, several studies have reported that restricted mobility is associated with an increased VTE risk in stroke patients [23,39,40]. Further, stroke patients frequently develop medical complications [41], such as infections [42], which can further enhance the high-risk situation. Indeed, patients with poststroke infections have been reported to have up to a 2- to 4-fold increased risk of VTE compared with stroke patients without infection [42,43]. These observations are in line with the findings from a previous population-based case-crossover study reporting that 68% of the total effect of stroke on VTE risk was mediated through immobilization and infection [8]. Both the degree and duration of immobility and the susceptibility to developing medical complications are closely related to stroke severity. Not surprisingly, measures reflecting stroke severity, such as a high score on the National Institutes of Health Stroke Scale, have been reported to be associated with increased VTE risk in several studies [[44], [45], [46], [47]]. We have previously shown that ischemic stroke was associated with a markedly higher risk of provoked VTE than unprovoked VTE and that immobilization and acute medical conditions were the main predisposing factors for stroke-related VTEs [2]. To reduce the burden of stroke-related VTE, further identification of risk factors is needed, particularly risk factors that, in combination with ischemic stroke (and stroke-related factors), have supra-additive effects on VTE risk. Two previous studies have assessed the impact of BMI on VTE risk in ischemic stroke patients using a prediction framework, and their results do not appear to be in line with our findings. Liu et al. [23] reported that stroke patients with a BMI of ≥25 kg/m^2^ had a 2-fold increased risk of DVT compared with stroke patients with BMI of <25 kg/m^2^. In a study of 92 stroke patients, Balogun et al. [24,25] reported a 3-fold increased risk of DVT per 10-unit increase in BMI, but the result did not reach statistical significance (*P* = .16). The distinct findings may be explained by differences in study design, characteristics of the study populations, and statistical modeling of BMI. Further, the clinical relevance of the reported associations in both studies is uncertain because outcomes were restricted to DVT only, and outcome assessment was conducted by a screening program (ie, a large proportion of asymptomatic events were included). In addition, obesity was not investigated according to the common clinical cutoff at BMI of ≥30 (WHO definition). In our study, we used the regular clinical cutoff for obesity, and only symptomatic VTE events were included. The main strengths of our study include the prospective study design with recruitment of participants from the general population, the long-term follow-up, and the objective validation of both ischemic stroke and VTE events. Moreover, the high attendance rate in the Tromsø Study and the wide age distribution of the participants increase the generalizability of the results. Some limitations merit attention. First, even though our study was derived from a large cohort, a low number of participants and VTE events in the joint exposure group(s) resulted in large CIs for the risk estimates and measures of biological interaction. Therefore, our findings must be interpreted with caution. Second, unfortunately, we did not have information on stroke severity, ischemic stroke subtypes, and use of pharmacologic thromboprophylaxis during the study period and could therefore not adjust for these possible confounders in multivariable analyses. Furthermore, as obesity is associated with AF and as AF is a risk factor for ischemic stroke, an increased VTE risk in obese stroke patients could potentially be masked by the use of anticoagulant treatment for stroke prevention in study participants with AF. However, adjustment for AF had minor impact on the risk estimates. Third, the classification of obese and nonobese study participants was based on BMI measured once at baseline. As these measures may be susceptible to change over time because of the long follow-up period, it could result in an underestimation of our risk estimates due to regression dilution bias. However, it has previously been shown in the Tromsø Study that risk estimates for VTE were similar in time-fixed analyses with single measurements of BMI and time-varying analyses with repeated measurements [48]. To conclude, the combination of ischemic stroke and obesity did not result in an excess risk of VTE, and obesity therefore does not appear to be essential for the risk assessment of VTE in patients with ischemic stroke.
