# Supplementary material for: Joint effect of ischemic stroke and obesity on the risk of venous thromboembolism: the Tromsø Study
Source: Res Pract Thromb Haemost. 2024 Mar 27;8(3):102392. doi: 10.1016/j.rpth.2024.102392 (PMC11043863; doi:10.1016/j.rpth.2024.102392)
Supplement: Supplementary material [file mmc1.docx]

**Supplementary Table S1** Crude incidence rates (IRs) and adjusted hazard ratios (HRs) with 95% confidence intervals (CIs) of provoked and unprovoked venous thromboembolism (VTE) according to ischemic stroke (IS) and obesity exposure: The Tromsø Study 1994-2014

|  |  |  |  |  | **Joint effects^¶^** | |  | **Within IS group^#^** | |
| --- | --- | --- | --- | --- | --- | --- | --- | --- | --- |
|  |  | **Person-years** | **VTE events** | **Crude IR^§^ (95% CI)** | **Model 1^*^  HR (95% CI)** | **Model 2**^†^ **HR (95% CI)** |  | **Model 1^*^  HR (95% CI)** | **Model 2**^†^ **HR (95% CI)** |
| **Provoked VTE** |  |  |  |  |  |  |  |  |  |
| IS - | Obesity - | 400 129 | 319 | 0.8 (0.7 - 0.9) | Reference | Reference |  |  |  |
| IS - | Obesity + | 46 745 | 77 | 1.6 (1.3 - 2.1) | 1.59 (1.24 - 2.05) | 1.64 (1.27 - 2.10) |  |  |  |
| IS + | Obesity - | 5 749 | 27 | 4.7 (3.2 - 6.8) | 2.24 (1.50 - 3.35) | 2.35 (1.57 - 3.51) |  | Reference | Reference |
| IS + | Obesity + | 1 291 | 10 | 7.7 (4.2 - 14.4) | 3.42 (1.81 - 6.46) | 3.84 (2.02 - 7.28) |  | 1.42 (0.68 - 2.96) | 1.49 (0.71 - 3.13) |
|  |  |  |  |  |  |  |  |  |  |
| **Unprovoked VTE** |  |  |  |  |  |  |  |  |  |
| IS - | Obesity - | 400 129 | 282 | 0.7 (0.6 - 0.8) | Reference | Reference |  |  |  |
| IS - | Obesity + | 46 745 | 78 | 1.7 (1.3 - 2.1) | 1.85 (1.43 - 2.38) | 1.92 (1.48 - 2.45) |  |  |  |
| IS + | Obesity - | 5 749 | 12 | 2.1 (1.2 - 3.7) | 1.07 (0.59 - 1.92) | 1.14 (0.63 - 2.05) |  | Reference | Reference |
| IS + | Obesity + | 1 291 | 2 | 1.5 (0.4 - 6.2) | 0.76 (0.19 - 3.06) | 0.87 (0.21 - 3.51) |  | 0.83 (0.18 - 3.79) | 0.98 (0.21 - 4.50) |

IS+/- indicates incident IS/no incident IS during follow up, respectively. Of note, IS was included as a time-varying risk factor in the Cox regression models. Obesity +/- indicates body mass index ≥/< 30 kg/m^2^ at baseline, respectively.
^§^Per 1000 person-years. ^¶^Joint effects of IS and obesity using non-obese subjects without IS as reference group. ^#^Impact of obesity in subjects with IS, using non-obese subjects with IS as reference group. ^*^Model 1: Age as time scale, adjusted for sex. ^†^Model 2: Model 1 + atrial fibrillation.

**Supplementary Table S2** Measures of biological interaction with 95% confidence intervals (CIs) for the joint effect of ischemic stroke and obesity (body mass index ≥30 kg/m^2^) for provoked and unprovoked venous thromboembolism (VTE): The Tromsø Study 1994-2014

|  | **Relative excess risk attributable to interaction (95% CI)** | **Proportion attributable to interaction (95% CI)** |
| --- | --- | --- |
| **Provoked VTE** | 0.85 (-1.72 to 3.43) | 0.22 (-0.32 to 0.77) |
| **Unprovoked VTE** | -1.17 (-2.63 to 0.28) | -1.35 (-4.74 to 2.03) |
